# Solar Output Controls Periodicity in Lake Productivity and Wetness at Southernmost South America

**DOI:** 10.1038/srep37521

**Published:** 2016-11-21

**Authors:** Marta Pérez-Rodríguez, Benjamin-Silas Gilfedder, Yvonne-Marie Hermanns, Harald Biester

**Affiliations:** 1 Departamento de Edafoloxía e Química Agrícola. Facultade de Bioloxía. Campus Vida. Universidade de Santiago, Santiago de Compostela, Spain; 2 Institut für Geoökologie, AG Umweltgeochemie, Technische Universität Braunschweig, 38106 Braunschweig, Germany; 3 Lehrstuhl für Hydrologie, Universität Bayreuth, Universitätsstr. 30, 95440 Bayreuth, Germany

## Abstract

Cyclic changes in total solar irradiance (TSI) during the Holocene are known to affect global climatic conditions and cause cyclic climatic oscillations, e.g., Bond events and related changes of environmental conditions. However, the processes how changes in TSI affect climate and environment of the Southern Hemisphere, especially in southernmost South America, a key area for the global climate, are still poorly resolved. Here we show that highly sensitive proxies for aquatic productivity derived from sediments of a lake near the Chilean South Atlantic coast (53 °S) strongly match the cyclic changes in TSI throughout the Holocene. Intra-lake productivity variations show a periodicity of ~200–240 years coherent with the time series of TSI-controlled cosmogenic nuclide ^10^Be production. In addition TSI dependent periodicity of Bond events (~1500 years) appear to control wetness at the LH site indicated by mineral matter erosion from the catchment to the lake assumingly through shifts of the position of the southern westerly wind belt. Thus, both intra-lake productivity and wetness at the southernmost South America are directly or indirectly controlled by TSI.

The recent climate between 41° and 53°S is mainly controlled by the southern westerly wind belt (SWW), which affects the southern ocean circulation and consequently the global climate^1^,^2^ and changes in seasonal insolation cause variations in the position and intensity of the SWW^3^,^4^,^5^,^6^. Moreover, precipitation at the hyper-humid western side of the Andes has been linked to solar activity through SWW shifting during the Late Holocene, although no cyclicity could be found^2^,^7^.

During the Holocene, one of the strongest effects on the Northern Hemisphere (NH) climate is attributed to the 1500 yrs cycle in solar output, known as Bond Cycles^8^,^9^. Bond Cycles lead to periodic cooling and variations in the production of North Atlantic deep water and have also affected biogenic productivity in terrestrial aquatic systems^10^. In the Southern Hemisphere several studies have associated abrupt centennial to millennial scale variations in the mid-latitude records with Bond events such as the South America monsoon system (SAMS), although the physical mechanisms linking both are yet unclear^11^,^12^,^13^,^14^.

Recently, the relevance of a 200–250 years periodicity in the Southern Hemisphere climate has been demonstrated. A stratigraphic record from Lago Cipreses in Southwestern Patagonia (51°S) reveals recurrent ~200 years positive Southern Annual Mode-like (SAM) phases (dry/warm) during the last three millennia^15^. The authors noted the common structure of centennial changes in SAM with paleoclimate records from the NH, and an in-phase interhemispheric coupling thought atmospheric teleconnections^15^. Similar, a recent study of enhanced westerly intensity during the last 2,600 years at the Falkland Islands (Patagonia Atlantic coast) show the same periodicity, coherent with radiocarbon production rates, suggesting that solar forcing plays a dominant role in modulating the strength of the SWW^16^. Moreover, a short (1,500 years) but high-resolution speleothem record from south-central Brazil showed the sensitivity of the SAMS to solar forcing with a cyclicity of 208 years.

All these studies are based on paleoclimate records reaching back in time for not more than 3,000 years, so no information of previous periods is yet available and the processes behind Holocene climatic changes associated with short-term cyclic variations in TSI are still unclear. One reason might be that high-resolution proxies, sensitive to TSI are not easily available for the southernmost SH because of the small landmass and the extreme climatic zonation of this key region for the ocean–atmospheric circulation system of southern mid to high latitudes.

Here, we report results from a multi-proxy (Fourier transform infrared spectroscopy (FTIR) and geochemical data) investigation of a 12,500 cal BP lake sediment record from Southern Patagonia (Strait of Magellan). We used multiple principal component analysis (PCA) to extract productivity indicators as well as climate-dependent chemical signals from the entire FTIR data and from the upper core section (~ 5,500 cal BP). Moreover, evaluation of Holocene TSI cyclicity has been based on ^10^Be concentrations derived from ice cores^17^ to evaluate the effect of TSI variations on aquatic productivity.

## Sedimentary and climatic history of Lago Hambre

**Figure 1:**
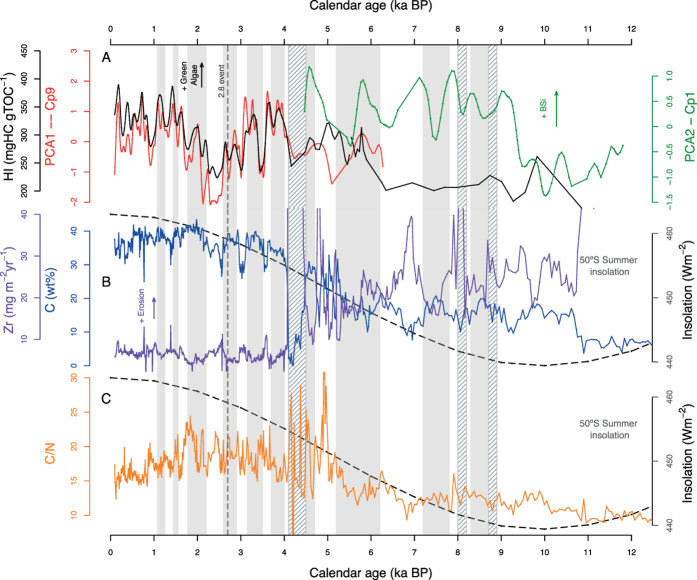
Records of sedimentary and climate proxies of Lago Hambre (LH). (**A**) Aquatic productivity proxies: Hydrogen index (HI)^24^, PCA1–Cp9 and PCA2–Cp1, were extracted from FTIR data (650 samples) by two separate Principal Component Analyses with varimax rotation using samples before 6.3 and after 4.5 yr BP, respectively. All data were smoothed. PCA2–Cp1 was shifted by ~200 years (see the text). (**B**) Carbon concentration and Zr accumulation record in LH and Holocene summer insolation (Dec-Feb) at 50 °S (dashed lines) from^27^. (**C**) Carbon/Nitrogen ratios and Holocene summer insolation at 50 °S. Grey bars indicate drier events at LH – derived from Zr accumulation (see Fig. 2) mentioned in the text. Streaked bars show tephra layers^46^. Bar at ~4,250 cal BP indicates coincidence of the tephra with a period of several short term erosion events. Bar line indicates the dry event at 2.8 kyr BP (i.e. ref. 38).

Lago Hambre (LH) (Fig. S1) is a small 17 m deep lake, located 50 km south of Punta Arenas (53°36′ 13.19″S, 70°57′ 8.77″ W). The lake lacks surface inflows, but gullies reflect intermittent surface inflow during periods of intense precipitation. The sedimentary and climatic history of LH is characterized by steadily increasing rates of organic carbon accumulation from the early Holocene until ~5,500 cal BP, when the record is interrupted by a series of intense erosion events (Fig. 1). These have been interpreted as storm events during a period of climate transition to warmer and wetter conditions^6^,^18^. A global set of environmental archives support a major global climate change between 5,600 and 5,000 cal BP e.g.^19^,^20^,^21^. Moreover, tephra layers at 4,250, 8,200, 8,800, and 14,300 cal BP further are intercalated within the sedimentary record, which are no direct indicators of climatic change.

In the past 4,000 cal BP, organic carbon accumulation in LH was generally higher and more variable than in previous periods (Fig. 1). Carbon/nitrogen ratios (C/N) increased from an average value of 8 to about 18, indicating organic matter (OM) sources, shifted from a dominance of algae-derived OM between 12,500 cal BP and 5,500 cal BP to increasing fluxes of terrestrial OM to the lake due to the development of *Nothofagus* forests in the lake’s catchment after 11,000 cal BP^22^ (Fig. 1). Beside the general trend of increasing organic carbon accumulation, a strong short-term variability in OM fluxes from the two different carbon sources becomes most pronounced in the past 4,500 cal BP (Fig. 1). Accumulation of eroded allochthonous minerogenic material (MM) is inversely related to that of carbon, and show a continuous decrease since about 11,000 cal BP until present. This general decrease in MM fluxes to the lake is mainly attributed to the development of the organic-rich forests soils in the past 4,500 cal BP which diminish erosion of MM.

## Productivity proxies from FTIR data

Most of the variance in the FTIR data extracted by PCA from the entire data set was explained by changes in OM- (58%, PCA-Cp1) and MM accumulation (40%, PCA-Cp2) (see supplementary discussion). Using the FTIR data of the past 5,500 cal BP, PCA1 extracted a signal (PCA1-Cp9) based on spectroscopic bands that are characteristic for aliphatic compounds associated with green algae (Figure S3). This specific spectrum is related to algaenan^23^, an insoluble, non-hydrolysable, and highly aliphatic macromolecule that serves as a structural component in the cell wall of freshwater green algae^23^. This component shows a significant (80%) cyclicity of 203 years since 5,500 cal BP (Fig. S4). Accordingly, positive loadings of PCA1-Cp9 indicate increased aquatic productivity. This interpretation is supported by a significant positive correlation (r = 0.74, n = 226) of the algaenan signal with the hydrogen index (HI) (Fig. 1), a proxy of the source of OM^24^, where high values of HI are typical for autochthonous OM^25^. PCA components extracted from FTIR data of sediments accumulated between 12,500 cal BP and 5,500 cal BP differed from those extracted from the past 5,500 cal BP. Most importantly, the signal of algaenan (PCA1-Cp9) was absent in the PCA components extracted from the data prior to 5,500 cal BP. Instead, a component, based on bands typical for biogenic silica (PCA2-Cp1) (Fig. S3) was detected indicating predominance of diatoms as primary producers.

At the end of the Pleistocene and early Holocene (~12,500 cal BP to ~11,000 cal BP), average C/N ratios below 10 indicate autochthonous productivity as the major source of OM in the sediments. Simultaneously, carbon accumulation and HI values both were very low indicating relatively little productivity and high OM decomposition, respectively (Fig. 1). Carbon accumulation and increasing BSi and HI values suggest that in-lake productivity increased from 11,000 to 6,800 cal BP followed by increasing fluxes of OM from the catchment until 5,500 cal BP (Fig. 1). Despite the general increase in terrestrial organic matter fluxes, in the upper part of the record, the C/N ratios and average HI values indicate a mix between contributions from aquatic productivity and terrestrial sources and oscillation in the prevalence of the two sources. The correlation between the two sources is negative (r = −0.64) supporting the interpretation of change of the major source: low C/N ratio and high HI values indicate greater proportion of aquatic (higher productivity) than terrestrial OM and vice versa. Changes in the prevalence of the OM source point to changes in the major controlling factors TSI, temperature, and wetness (precipitation). No positive correlation between productivity proxies (PCA1-Cp9, HI and PCA2-Cp1) and inputs of inorganic matter from the catchment could be found, which excludes nutrient fluxes as the major driver behind changes in intra-lake productivity. Moreover, no trends or cyclic fluctuations in temperature in the area during the Holocene have been reported^26^ which could explain the observed changes in intra-lake productivity. Thus, the variability in OM accumulation and especially in proxies indicating changes in the intensity of aquatic production, algaenan (PCA1-Cp9) and BSi, (PCA2-Cp1) imply that the cyclic changes in solar insolation must be the major factor driving these changes similar to what has been observed in lakes in the Arctic^10^.

## Solar insolation changes and long-term aquatic productivity

When the long–term development of summer solar insolation at 50°S^27^ is compared to C/N ratios, PCA2-Cp1 (BSi) and carbon accumulation between 12,000 and 5,500 cal BP it becomes evident that summer (Dec-Feb) insolation is the main driver of long-term productivity in Lago Hambre (Fig. 1). After 5,500 cal BP, the generally high accumulation of OM in the sediments is attributed to a further increase in summer insolation as well as wetter and warmer conditions favouring both aquatic productivity and fluxes of catchment-derived OM (Fig. 1).

The long-term development of summer insolation from low values during the early- to higher values during the middle- and late Holocene (Fig. 1) also explains the long-term evolution of the two different signals for aquatic productivity (algaenan (PCA1-Cp9) and BSi (PCA2-Cp1)) extracted from the FTIR data. Because green algae require higher irradiance to grow than most cyanoprokaryotes and diatoms, they generally possess a higher photosynthesis saturation parameter I_k_. Accordingly, lower insolation during early- and mid-Holocene at 50°S favours diatoms (BSi) over green algae abundance and vice versa during the past 5,500 cal BP, when insolation was generally higher (Fig. 1).

**Figure 2:**
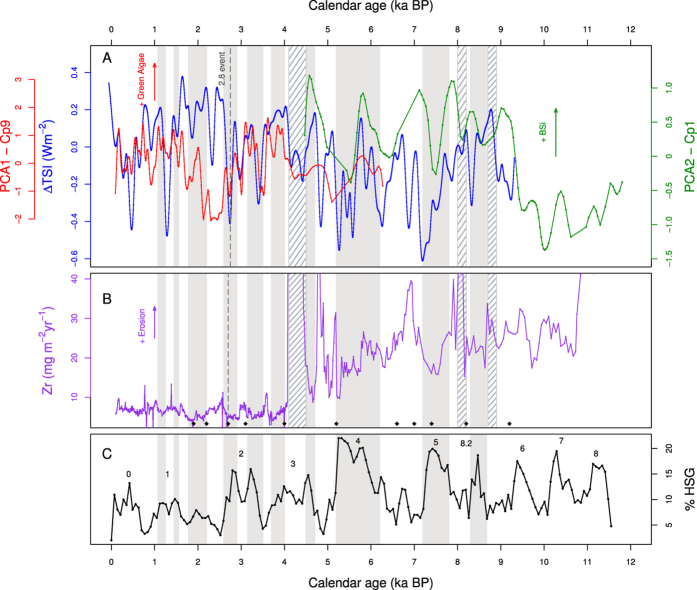
Proxy records of aquatic productivity, mineral matter sedimentation and solar activity. (**A**) ^10^Be-based reconstruction of total solar irradiance (ΔTSI) from^17^. Aquatic productivity proxies in LH sediments: PCA1–Cp9 and PCA2–Cp1 (see Fig. 1). (**B**) Erosion events derived from zirconium (Zr) accumulation rates in LH. Black dots indicate maximum precipitation in SAMS^14^ (**C**) Bond events shown as percentage of Hematite-stained quartz grain (%HSG) as recorded from North Atlantic marine core VM29-191^9^. Codes of the bars are the same as Fig. 1.

Changes in averaged total solar irradiance (ΔTSI)^17^ show the same trend than changes in aquatic productivity as indicated by the intensity of the algaenan (PCA1-Cp9) and BSi (PCA2-Cp1) signals (Fig. 2). This relationship is pronounced during the past 4,500 cal BP when summer insolation was generally high and green algae were dominant. However, during some periods (3,000–2,000 cal BP and 5,500 to 4,500 cal BP), this relationship appears to be partly overwritten by regional or local erosion events and becomes blurred. Between 9,300 cal BP and 5,500 cal BP, the relation between aquatic productivity (BSi, PCA2-Cp1) and ΔTSI remains, but is less pronounced due to the lower resolution in the lower core section. Additionally, the BSi (PCA2-Cp1) and ΔTSI records seem to show an offset of ~200 years, probably due to the low resolution of the BSi-data at this core section. However this ~200-year offset is within the 2σ uncertainty of the calibrated ^14^C dates, why we shifted the data here by 200 years

Spectral analyses of productivity proxies reveal significant periodicities of 203 years for PCA1-Cp9 and 201 and 240 years for HI (see S4). Both periodicities have been previously observed in records of the TSI controlled records of atmospheric ^14^C and ^10^Be^28^,^29^, which points to a relation between intra-lake productivity in LH and the Vries solar cycle^30^. Previous studies reported this periodicity for productivity records in the Southern Ocean^31^,^32^.

## Humidity in Lago Hambre is linked to Total Solar Irradiance and Bond events

Besides the effect of TSI on aquatic productivity, cyclic variations in sediment composition also suggest changes in climate conditions at the Strait of Magellan during periods of low or high solar insolation.

The spectral analyses of Zr accumulation (erosion proxy) in the LH sedimentary record reveal significant periodicities at 1878, 939, 250 and 209 years (Fig. S4). These periodicities can be related to two climatic time scales - centennial to millennial (939–1878 years) and centennial changes (209–250 years).

As mentioned in the previous section, the existence of a 200–250 periodicity in the sediment data has been associated with solar cycles of de Vries-Suess^30^ and identified in numerous, globally distributed Holocene climate records^33^,^34^. However, the role of this solar cyclicity as a major driver of the climate in the southernmost Southern Hemisphere is still under debate. For example, it has been recently shown that the monsoon in Central-east Brazil (Minas Gerais state) has a pronounced relation with solar Vries cycles, at least in the last 1500 yrs^35^.

For southern Patagonia, the predominant factor influenced by changes in TSI is most likely the shifting^7^ and the strength^16^ of the SWW at the western coast of southern Chile. Here, periods of lower solar activity are correlated with wetter conditions and stronger winds at the northern part of the SWSW-belt (41°S), while the weaker winds produce dryer conditions at 53°S^6^. Comparing the TSI record with Zr accumulation record (Fig. 2) indicates that periods of high TSI (and productivity) largely correspond to periods of low mineral matter fluxes indicating relatively dry conditions (see for example isolated events at 450, 1,300, 2,400, 2,700, 3,400, 5,200, 6,300 or 7,200 cal BP) due to weaker SWW at 53°S. However, this is not consistent throughout the entire record, because local climatic changes in temperature or precipitation may influence MM fluxes to the lake independent from the cyclic changes on the hemisphere scale.

A recently published study on a Falkland Islands peat sequence (51°S) support our interpretation of a solar control on SWW. Here, a record of charcoal concentrations in peat, which represents atmospheric charcoal fluxes from southern Patagonia reveal a pervasive 250-years periodicity in SWW intensity coherent with the TSI controlled production of ^14^C during the last 2,600 years^16^. The authors identified several distinct charcoal concentration peaks which they attributed to stronger SWW and drier conditions causing more frequent forest- and peatland fires in southern Patagonia.

Although spectral analyses of both records (LH and Falkland Islands) show the same periodicity and a clear relation with SWW, periods of maximum SWW intensity and their effect on humidity is not the same. Like on Annekov Island (South Georgia)^36^ and at windward sites of the Andes^6^ the increase in SWW at LH imply wetter conditions, while the reverse was observed on the Falkland Island^16^. On the other hand, the Falkland charcoal record indicates that the central Southern Hemisphere westerly winds were particularly strong between 2,000 and 1,000 cal BP^16^. Although the LH record shows several erosion events during this time, we cannot consider it a period of particularly strong erosion, especially when compared to the early or middle Holocene. Only the prominent peak of SWW-intensity at 1,000 cal BP corresponds to one of our erosion events at LH. Other Holocene paleo-climatic records from southernmost Patagonia support our findings^37^. Although no cyclicity in the changes of wet and dry periods were found in those studies, probably due to insufficient sensitivity of the sampling sites or the archives, three of the wet events detected in the LH-record (~3,600, 3,090, 2,250 and ~1,350 cal BP) were congruent with those detected in a coeval stalagmite (MA1) from the super-humid Andes^37^. In addition, regional distributed dry periods at 2,700 cal BP^38^ and at ~4,500 cal BP^37^,^39^,^40^,^41^, deduced from geochemistry, vegetation- or pollen distribution changes in Southern Patagonia, correspond to periods of high aquatic production and drier conditions at LH.

Centennial to millennial-scale variations^9^ in solar forcing may be responsible for the ~900 to ~1,800 years cycles in LH erosion events, through changes in the North Atlantic Meridional Overturning Circulation (AMOC), which is also considered being a main driver for the SAMS^14^,^35^. An atmospheric teleconnection between the Intertropical Convergence Zone (ITCZ) and the SWW^42^ could connect precipitation at Lago Hambre with cooling of the North Atlantic, implicating a more complex control on wetness at the LH site.

When comparing the intensity of the MM signal (Zr accumulation rates) in LH sediments to %HSG (Bond events), drier conditions (weaker MM signals) appear to correspond to higher percentage of HSG (cooler condition in the northern Atlantic) and vice versa (Fig. 2). This relationship is in opposite to the one found by Stríkis *et al.*^14^, in speleothems in central-eastern Brazil (Minas Gerais state), where the abrupt increase in South American monsoon (higher precipitation) correlates positively with lower sea-surface temperatures in the North Atlantic during Bond events. Five of the wet periods (including the event at 8,200 cal BP), defined by Strikis *et al.*^14^, are recorded as dry events at LH (Bond events: 1,2,4,5 and 8.2). Compared to central-eastern Brazil, the relationship between dryness at LH and Bond events is more pronounced in the middle and early Holocene (events 5 and 8.2). During event 4, the record shows lower precipitation than earlier or later periods, however the general decrease in erosion clearly reflects the general decrease in precipitation from the middle to the late Holocene in Southern Patagonia^6^. The relationship between dry conditions at LH and wet (cold) in central-eastern Brazil (North Atlantic) is particularly pronounced during Bond event 2 which is sufficiently resolved to reflect a decrease in humidity (and temperature) at ~3,000 cal BP in the middle of the event (Fig. 2). On the other hand, the maximum of the abrupt precipitation events in Minas Gerais^14^, do not agree with the events at LH (Fig. 2). This could indicate that there is no direct causal link, but a common driver.

The correlation between wetter periods at LH and wetter periods at the western side of the Andes^37^ suggests a link between LH humidity conditions and the intensity of SWW and thus solar influence on sea surface temperatures of the eastern South Pacific Ocean which have been suggested to control shifts in the southern westerly wind belt^6^ at longer time scales. Unlike the observed direct relationship between primary aquatic productivity and TSI, it remains unclear how solar changes affect the SWW at 53 °S. The connection between climatic conditions at LH and North Hemisphere climatic changes during the Holocene suggest that mechanism residing in the atmosphere such as changes in the Hadley cell circulation^43^, solar variability^9^, or coupled ocean–atmosphere models^44^,^45^, are likely to play and important role here.

## Methods

### Location and Sampling

Lago Hambre (Fig. S1) is located 50 km south of Punta Arenas, near the Strait of Magellan, in southernmost Patagonia, Chile (53°360 13.1900 S, 70°570 8.7700 W). It is situated about 80 m a.s.l. and is a small lake with a surface area of 13,700 m^2^ and a maximum depth of 17 m. The catchment-to-lake ratio is about eight. The lake is situated in the zone of sub-Antarctic deciduous forest with *Nothofagus pumilio* and *Nothofagus antarctica.*

A long sediment core was recovered from the deepest part of the lake in 2008 using a 5-m-long piston corer. The core was recovered in three single drives, with an offset of about 90 cm to ensure overlaps. Sediment cores were stored under dark and cool (4 °C) conditions in the laboratory before they were split lengthwise using a stainless steel saw. Sub-samples were taken at 1 cm intervals in the upper 470 cm of the piston core, and at 2-cm intervals below 470 cm.

Concentrations of carbon (C) and nitrogen (N) in the carbonate-free samples were determined by GC-TCD after thermal combustion in an elemental analyzer (Euro EA3000, Eurovector, Germany). Samples were analyzed for zirconium (Zr), using an energy-dispersive XRF mini-probe multi-element analyzer (EMMA), see refs 24 and 46.

The hydrogen index (HI) i.e. the quantity of pyrolyzable hydrocarbons (S2) per gram TOC (mg HC gC^−1^), was determined by means of a Rock–Eval-II-plus-S3-unit at the Alfred Wegener Institut für Polar- und Meeresforschung in Bremerhaven, Germany, using sample aliquots of 30–50 mg.

### Age Model

The age-depth model is based on 18 Accelerator Mass Spectrometry (AMS) ^14^C dates on terrestrial macro-fossils, i.e. leaves and wood, as well as a tephra layer of known age (Mt Burney, 4,250 cal yr BP, ref. 39). For a more detailed description of the age model see refs 24 and 46 and supporting information.

### Infrared Spectroscopy (IR)

A total of 650 samples were analysed by Infrared Spectroscopy (IR). FTIR spectra of freeze-dried and ground samples of lake sediment were obtained using a Vector 22 FTIR spectrometer (BrukerOptik, Ettlingen, Germany) in absorption mode, with subsequent baseline subtraction on KBr pellets (200 mg dried KBr and 2 mg sample). Measurements were recorded from 4,500 to 300 cm^−1^ using a resolution of 2 cm^−1^. Thirty-two scans were taken per sample and averaged to obtain the final spectra. In order to improve the comparison between samples, each FTIR spectrum was re-scaled to relative absorbance (i.e. dividing the absorbance value of each point of the spectrum by the summed values of the analysed spectral region).

### Statistical Modelling

Principal component analysis (PCA) was performed on the IR data to identify the main chemical signals of the lake core and relate them to underlying environmental processes in the lake and its catchment. To maximize the loadings of the original variables, a varimax rotation was applied. In this study, we performed the PCA on a transposed matrix (samples as columns and absorbance bands as rows). In contrast to PCA on direct matrices, that on the transposed matrix provides scores for the spectral bands and loadings for the samples. Each extracted component is represented by a spectrum of scores, which permits to identify the most relevant signals characterizing the composition of the samples, whereas sample’s loadings (or their squared values) indicate the weight of the identified compounds on the samples’ spectroscopic signal (related to its abundance in the samples).

We performed a PCA using all samples. In addition, due to the large compositional differences in the sediment along the core found in previous studies^22^,^30^, we also analyzed the main two sections separately: PCA1 for the samples between 94–6,273 cal BP and PCA2 for the samples 4,591–15,445 cal BP.

### Spectral analysis

The spectral analysis (REDFIT) of PCA1-Cp9, HI and Zr accumulation were performed with Past^47^ software. PCA statistical analyses were performed using the statistic environment R 3.1.1^48^.

## Additional Information

**How to cite this article**: Pérez-Rodríguez, M. *et al.* Solar Output Controls Periodicity in Lake Productivity and Wetness at Southernmost South America. *Sci. Rep.* **6**, 37521; doi: 10.1038/srep37521 (2016).

**Publisher’s note:** Springer Nature remains neutral with regard to jurisdictional claims in published maps and institutional affiliations.

## Supplementary Material

Supplementary Information
