# Supplementary material for: Solar Output Controls Periodicity in Lake Productivity and Wetness at Southernmost South America
Source: Sci Rep. 2016 Nov 21;6:37521. doi: 10.1038/srep37521 (PMC5116613; doi:10.1038/srep37521)
Supplement: Supplementary Information [file srep37521-s1.pdf]

Supplementary Information

Solar Output Controls Periodicity in Lake Productivity and Wetness at Southernmost South America

Marta Pérez-Rodríguez[1,2*], Benjamin-Silas Gilfedder[3], Yvonne-Marie Hermanns[2], Harald Biester[2*]

[1]Departamento de Edafoloxía e Química Agrícola. Facultade de Bioloxía. Campus Vida. Universidade de Santiago, Santiago de Compostela, Spain.

[2]Institut für Geoökologie, AG Umweltgeochemie, Technische Universität Braunschweig, 38106 Braunschweig, Germany.

[3]Lehrstuhl für Hydrologie, Universität Bayreuth, Universitätsstr. 30, 95440 Bayreuth, Germany.

*Correspondance to: *mperez.rodriguez@usc.es, *h.biester@tu-bs.de

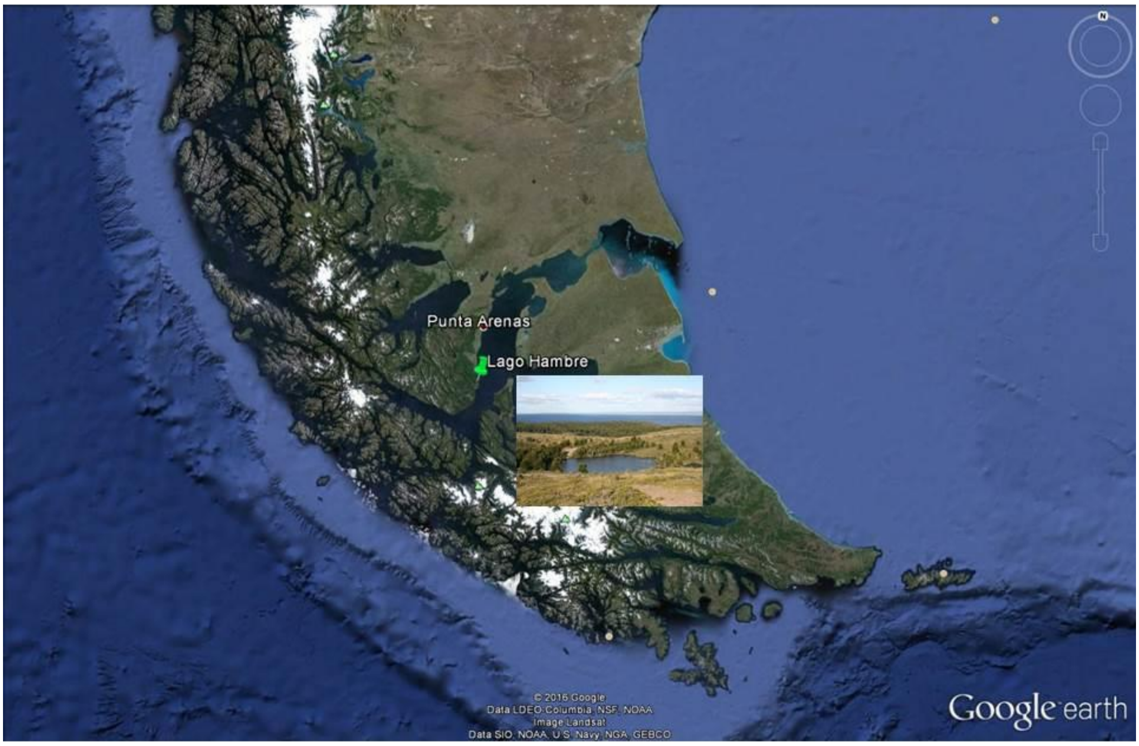

Figure S1: Map of southernmost South America and location of Lake Hambre, Chile (53 °S). Map by Google, photo of LH by Y.-M. Hermanns.

Table S1. Radiocarbon dating of Lago Hambre sediments performed on Nothofagus leaves and wood samples[1].

Laboratory	Calibration data set	Sample material	LAB ID	Depth (cm)	^{14}C yr BP	cal yr BP	2σ range (cal yr BP)
Keck, Irvine	SHcal04	Leaf	73893	67–68	235 ± 15	193	298–152
Keck, Irvine	SHcal04	Leaf	73894	92–93	945 ± 20	801	904–741
Keck, Irvine	SHcal04	Leaf	79254	136–137	1205 ± 15	1047	1168–980
Keck, Irvine	SHcal04	Leaf	79255	152–153	1220 ± 15	1080	1171–1037
WHOI, Woods Hole	SHcal04	Leaf	74832	200	1870 ± 30	1755	1862–1626
Keck, Irvine	SHcal04	Leaf	79256	255–256	2045 ± 15	1939	1993–1886
Keck, Irvine	SHcal04	Leaf	73895	325–326	2575 ± 15	2593	2740–2492
Keck, Irvine	SHcal04	Leaf	79257	412–413	3105 ± 15	3281	3354–3171
Keck, Irvine	SHcal04	Leaf	79258	554–556	4520 ± 15	5169	5286–4974
Keck, Irvine	SHcal04	Leaf	73896	632–634	5510 ± 15	6250	6300–6209
Keck, Irvine	SHcal04	Leaf	79259	722–724	6425 ± 15	7300	7417–7253
Keck, Irvine	SHcal04	Leaf	79260	804–806	7370 ± 20	8109	8182–8032
Keck, Irvine	SHcal04	Leaf	79261	926–928	8745 ± 20	9625	9730–9548
WHOI, Woods Hole	SHcal04	Wood	73016	1054	9360 ± 45	10504	10656–10297
Keck, Irvine	*INTCAL 09*	*Wood*	*79262*	*1162–1164*	*10595 ± 25*	*12622*	*12697–12579*
Keck, Irvine	*INTCAL 09*	*Wood*	*79263*	*1228–1230*	*12525 ± 25*	*14778*	*15157–14474*
WHOI, Woods Hole	*INTCAL 09*	*Wood*	*69372*	*1374–1376*	*13500 ± 60*	*16730*	*16944–16439*

Ages in italics were calculated with the Northern Hemisphere Calibration Curve INTCAL09 and 56 years were added according to McCormac et al. (2004)[2].

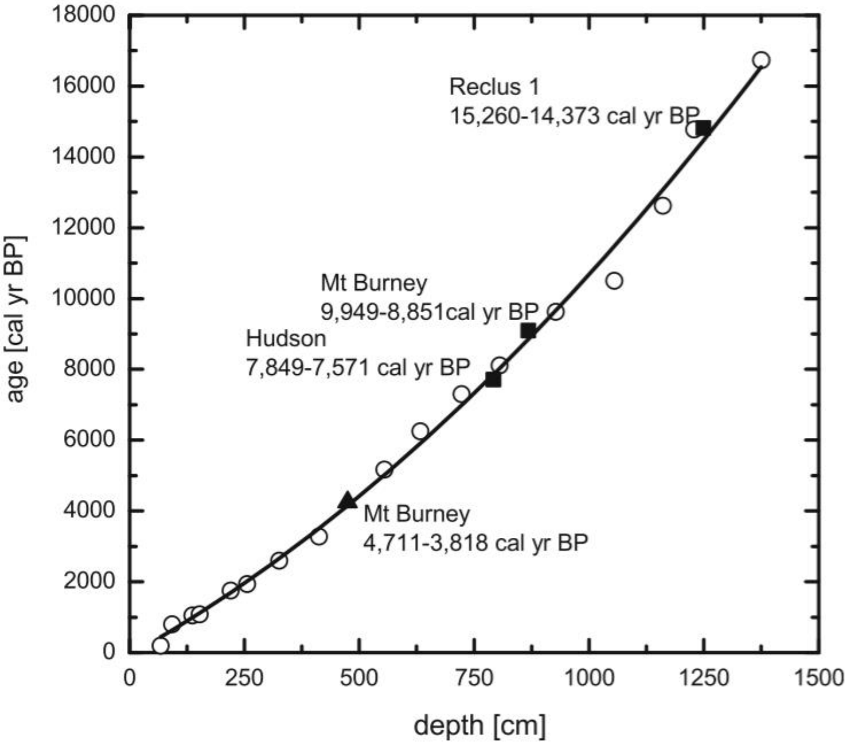

Fig S2. Age-depth model (2nd order polynominal) of the Lage Hambre piston sediment core based on the data in table S1. Open circles represent calibrated ^{14}C ages. The black triangle represents the tephra of the Mt Burney eruption 4,250 cal BP, which was included in the model. Black squares represent tephra layers, which are assumed to reflect the indicated eruptions of the Hudson, Mt Burney and Reclus volcanoes.

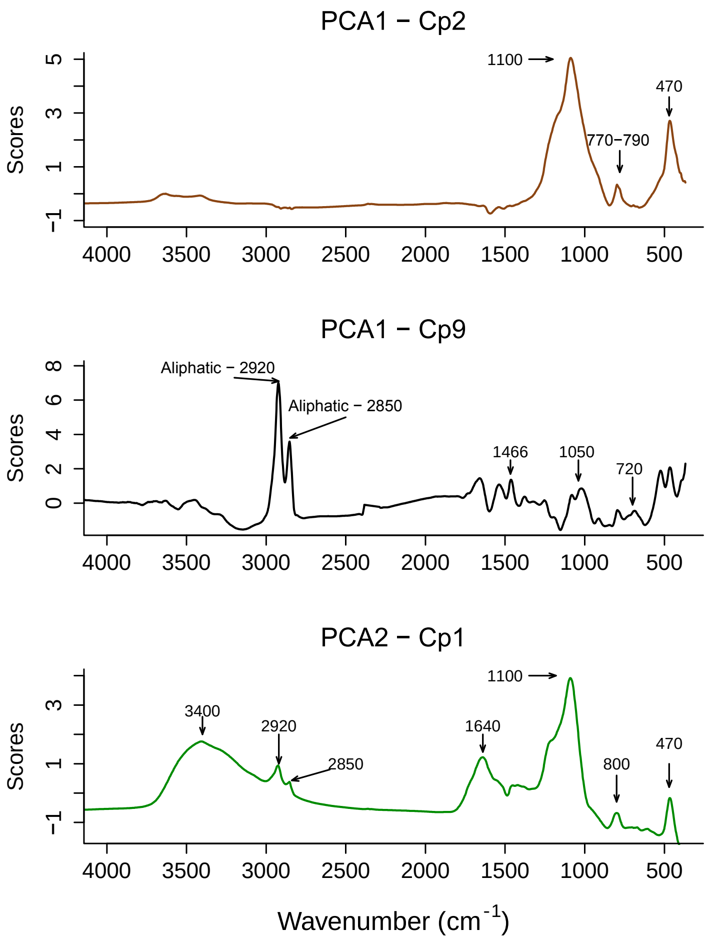

Fig S3: PCA-scores including characteristic peaks/bands of the main principal components extracted from the FTIR spectra.

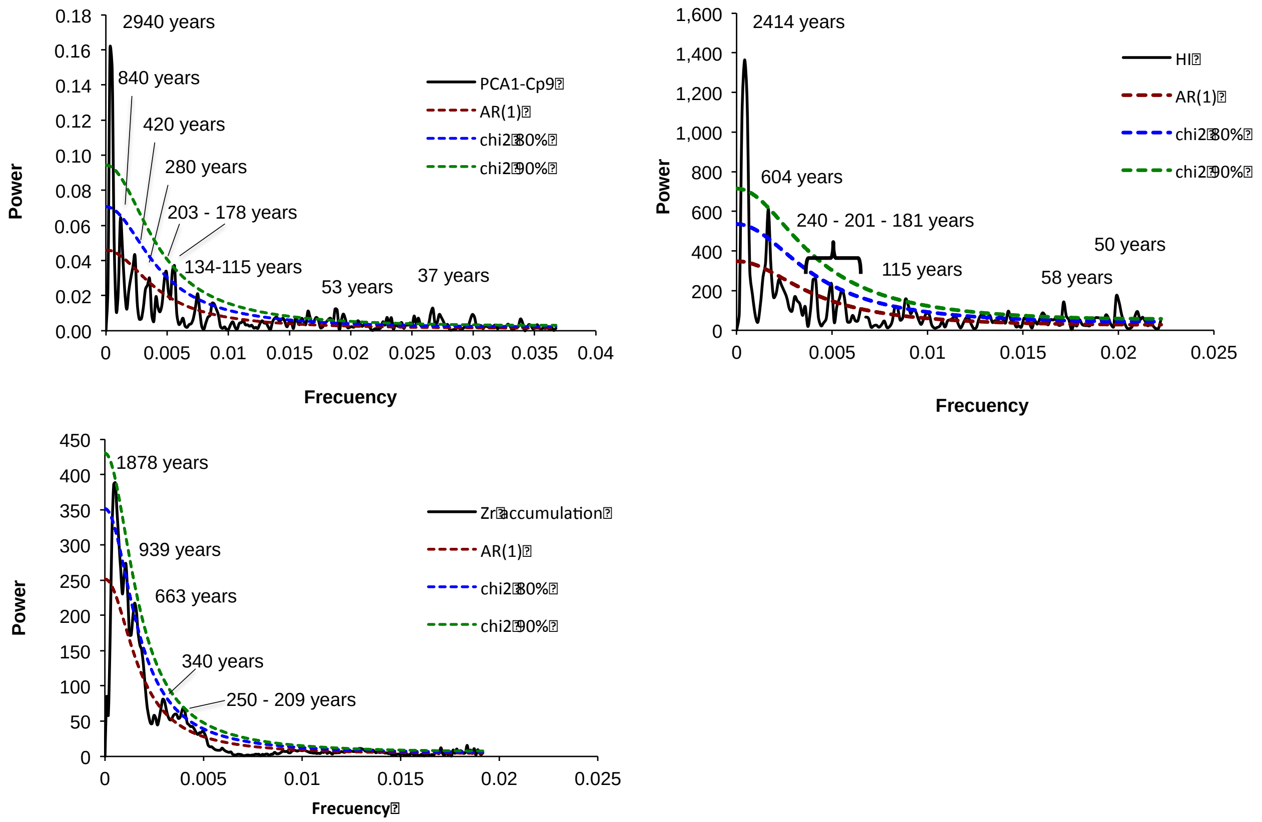

Fig S4: Spectral analysis (REDFIT[3]) of PCA1-Cp9, HI and Zr accumulation. Periodicities are shown in years. The analysis was made with Past software [4]. Chi-squared confidence levels at 80% and 90% are indicated.

Table S2 Main bands of FTIR spectra and association to functional groups in LH sediments

Bands	Assignment	Reference
470	Si- O-Si bonding vibrations	[5,6]
720	CH_2 wag; Long chain (>C4) alkanes	[6]
770-790	Quartz	[7]
800	Symmetric Si-O-Si stretching vibrations	[5,6]
1050	Ether bonds or hydroxilic groups	[9]
1100	Si-O-Si stretching vibration	[6,7]
1466	Aliphatic	[9]
1640	Secondary amines	[10]
2850	Aliphatic	[5,6]
2920	Aliphatic	[5,6]
3400	OH stretching	[5,6]

Statistical modelling interpretation

The PCA executed using all samples of the entire core extracted three components explaining 99.1% of the variance. The first component (Cp1) accounts for 57.6% of the total variability. It is characterized by high and positive values at 1,080, 1,640, 2,920 and ~3,300 cm^{-1} as well as medium and positive values at 1,420, 1,450 and 2,820 cm^{-1}. The second component (Cp2) accounts for 40.4% of the variability and is characterized by high positive values at 470 and 1,100 cm^{-1} as well as a double peak at 770 – 790 (800) cm^{-1}.

The interpretation of the geochemical components was based on their scores that represent their characteristic FTIR–spectra and the vertical distribution and correlation with the chemical elements measured. The first component has higher and positive bands characteristic for organic matter (OM): both fresh OM, represented by polysaccharides (peaks at 1,080 $cm^{-}1$, in covariation with OH signal at 3,300-3,400) and more decomposed OM (1,240 – 2,920 cm^{-1}). This is in agreement with the strong positive correlation of this component with carbon and nitrogen (r= 0.93 and 0.79), a moderate positive correlation with C/N (r=0.71) and a strong negative correlation with mineral proxies, such as zirconium (r=-0.78). The second component is characterized by positive peaks related to inorganic matter, such as clay minerals and silicates (silicon groups, Table 1). This interpretation is supported by the significant negative correlation of this component with carbon (r=-0.90), nitrogen (r=-0.73) and C/N ratios

(r=-0.61) and a significant positive correlation with zirconium (r=0.70). Thus, the first two components have been interpreted as total organic matter and total inorganic matter in the sediments. As expected, Cp1 and Cp2 are highly and negatively correlated (entire record).

The first three components of PCA1 are similar to those obtained by PCA using the entire data set, but with a different percentage of variance 56.0, 39.2 and 4.3%, respectively. Component 9 (Cp9) accounts for less than 1% of the data set variance. It shows high positive peaks at 2,850 and 2,920 cm^{-1} and moderate positive peaks at 720, 1,050 and 1,466 cm^{-1} (Fig. S2 and Table S1). Cp9 loadings correlate strongly with HI (r=0.74) and moderately with N (r=0.41). Based on findings by Blokker et al.[9], this component was identified to indicate a degraded compound of algeanan.

The first component of PCA2 (PCA2 – Cp1) accounts for 50.2% of the total variance. It scores highly positive at 1,100, 1,640, 2,850, 2,920 and 3,400 cm^{-1} and peaks at 470 and 800 cm^{-1} (Fig. S2). Three of these peaks (1,100, 800 and 471 cm^{-1}) were identified as distinct absorption bands of biogenic silica (BSi), which are associated with asymmetric Si-O-Si stretching, symmetric Si-O-Si stretching, and Si-O-Si bending vibrations, respectively (e.g.[5]). No positive peaks were found for bands at 470 and 800 cm^{-1} probably indicating other silicates (e.g. feldspar) absorbing on these bands of the IR spectrum (e.g. [5]). In addition, Cp1 also show other peaks that are commonly statistically related to BSi, e.g. aliphatic signals at 2,850 and 2,920 cm^{-1} [5]

References

1. Hermanns, Y. M. & Biester, H. A 17,300-year record of mercury accumulation in a pristine lake in southern Chile. *J. Paleolimnol.* **49,** 547–561 (2013)
2. McCormac FG, Hogg AG, Blackwell PG, Buck CE, Higham TFG, Reimer PJ (2004) SHCal04 southern hemisphere calibration 0–11.0 cal kyr BP. Radiocarbon 46:1087–1092
3. Schulz, M. & Mudelsee, M. REDFIT: Estimating red-noise spectra directly from unevenly spaced paleoclimatic time series. *Comput. Geosci.* **28,** 421–426 (2002).
4. Hammer, Ø., Harper, D. A. T. & Ryan, P. D. Paleontological Statistics Software: Package for Education and Data Analysis. *Palaeontol. Electron.* (2001).
5. Meyer-Jacob, C. *et al.* Biogeochemical variability during the past 3.6 million years recorded by FTIR spectroscopy in the sediment record of Lake El’gygytgyn, Far East Russian Arctic. *Clim. Past* **10,** 209–220 (2014).
6. Rosén, P. *et al.* Universally applicable model for the quantitative determination

of lake sediment composition using fourier transform infrared spectroscopy. *Environ. Sci. Technol.* **45,** 8858–65 (2011).

7. Ibarra, J., Muñoz, E. & Moliner, R. FTIR study of the evolution of coal structure during the coalification process. *Org. Geochem.* **24,** 725–735 (1996).
8. Rossel, R. A. V. & Behrens, T. Using data mining to model and interpret soil diffuse reflectance spectra. *Geoderma* **158,** 46–54 (2010).
9. Blokker, P. *et al.* Chemical structure of algaenans from the fresh water algae Tetraedron minimum, Scenedesmus communis and Pediastrum boryanum. *Org. Geochem.* **29,** 1453–1468 (1998).
10. Coates, J. P. The Interpretation of Infrared Spectra: Published Reference Sources. *Appl. Spectrosc. Rev.* **31,** 179–192 (1996).
